# An Optimized RNA Extraction Method From Micro-quantities of Guinea Pig Cartilage and Synovium for Osteoarthritis Research

**DOI:** 10.21769/BioProtoc.5348

**Published:** 2025-06-20

**Authors:** Nidhi Bhardwaj, Diksha Rana, Jyotdeep Kaur

**Affiliations:** Department of Biochemistry, Postgraduate Institute of Medical Education and Research, Chandigarh, India;

**Keywords:** Cartilage, Guinea pig, Osteoarthritis, RNA isolation, Synovium

## Abstract

Osteoarthritis (OA) is the primary cause of joint impairment, particularly in the knee. The prevalence of OA has significantly increased, with knee OA being a major contributor whose pathogenesis remains unknown. Articular cartilage and the synovium play critical roles in OA, but extracting high-quality RNA from these tissues is challenging because of the high extracellular matrix content and low cellularity. This study aimed to identify the most suitable RNA isolation method for obtaining high-quality RNA from microquantities of guinea pig cartilage and synovial tissues, a relevant model for idiopathic OA. We compared the traditional TRIzol^®^ method with modifications to spin column–based methods (TRIspin-TRIzol^®^/RNeasy^TM^, RNeasy^TM^ kit, RNAqueous^TM^ kit, and Quick-RNA^TM^ Miniprep Plus kit), and an optimized RNA isolation protocol was developed to increase RNA yield and purity. The procedure involved meticulous sample collection, specialized tissue processing, and measures to minimize RNA degradation. RNA quality was assessed via spectrophotometry and RT–qPCR. The results demonstrated that among the tested methods, the Quick-RNA^TM^ Miniprep Plus kit with proteinase K treatment yielded the highest RNA purity, with A_260:280_ ratios ranging from 1.9 to 2.0 and A_260:230_ ratios between 1.6 and 2.0, indicating minimal to no salt contamination and RNA concentrations up to 240 ng/μL from ⁓20 mg of tissue. The preparation, storage, homogenization process, and choice of RNA isolation method are all critical factors in obtaining high-purity RNA from guinea pig cartilage and synovial tissues. Our developed protocol significantly enhances RNA quality and purity from micro-quantities of tissue, making it particularly effective for RTqPCR in resource-limited settings. Further refinements can potentially increase RNA yield and purity, but this protocol facilitates accurate gene expression analyses, contributing to a better understanding of OA pathogenesis and the development of therapeutic strategies.

Key features

• Enables efficient RNA isolation from small, individual cartilage samples, eliminating the need for pooling and requiring minimal laboratory equipment.

• Provides a reliable and cost-effective method for obtaining high-quality RNA suitable for RT–qPCR and gene expression analysis, from challenging tissue types like cartilage.

Graphical overview

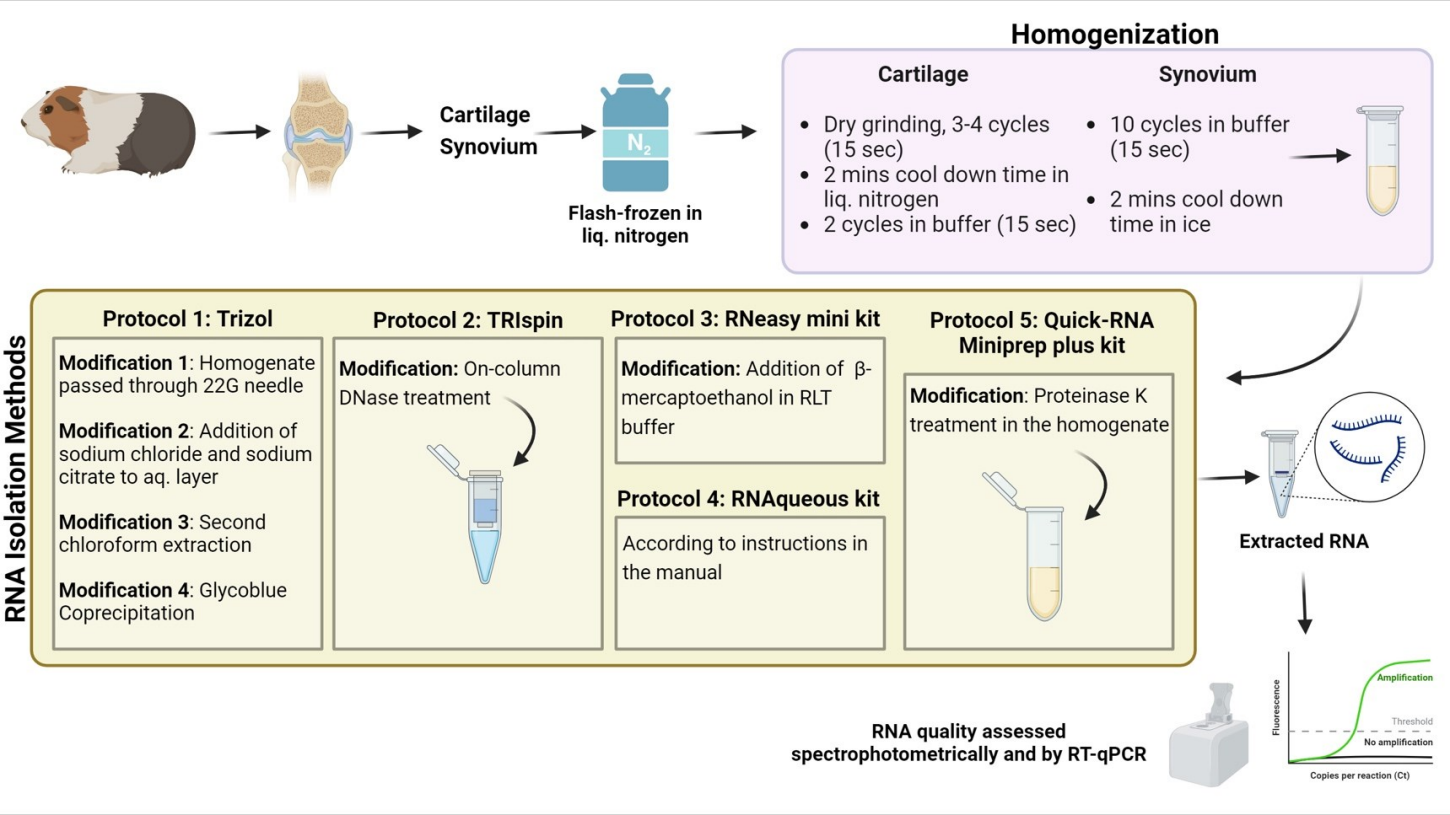

Graphical representation comparing modified RNA isolation protocols for efficient RNA extraction from guinea pig cartilage and synovium

## Background

Osteoarthritis (OA) is the prevailing cause of joint impairment, particularly in the knee joint, and is characterized primarily by inflammation, redness in the affected joints, persistent joint pain, and restricted movement. Based on the Global Burden of Disease Study 2019, research from 1990 to 2019 revealed a substantial increase in the prevalence of OA in specific joints. The prevalence of OA cases increased significantly by 113%, increasing from 247 million in 1990 to 527 million in 2019, with knee OA playing a major role in contributing to the overall burden [1]. Therefore, there is a compelling need to gain deeper insights into the pathological mechanisms underlying OA, and novel approaches, methodologies, and techniques must be employed to elucidate the pathogenesis of OA and to further explore therapeutic strategies. Articular cartilage, which is the most affected tissue in osteoarthritic conditions and undergoes degenerative disease, has a distinctive structural composition and consists of a very sparse distribution of chondrocytes surrounded by copious amounts of extracellular matrix (ECM) [2]. The majority of articular cartilage is composed of ECM, which consists mainly of glycosaminoglycans, collagens, and proteoglycans [3]. Moreover, the gradual deterioration of the articular surfaces in OA results in a significant decrease in the amount of cartilage and, consequently, in the amount of available tissue. This adds to the existing challenges faced while isolating RNA from cartilage tissues and underscores the importance of devising an appropriate methodology for procuring high-quality RNA from such tissues. In addition, articular cartilage is relatively hard, making it even more difficult to homogenize for RNA extraction without specialized equipment. To date, some of the reported methods for isolating RNA from articular cartilage are suitable only for larger cartilage samples weighing at least 100 mg, or they require the use of freezer-mills or microdisembranators [4,5] for homogenization, which are not readily available in all laboratories. The technique of RNA extraction from cartilage by first isolating chondrocytes [6,7] has certain drawbacks, as it can lead to the loss of heterogeneous chondrocyte populations in the cartilage and can be stressful for the cells, leading to a skewed representation of the gene expression profile. In addition, this is also a time-consuming process, especially when dealing with a large number of samples, which can lead to delays in downstream analysis. Protocols for the isolation of RNA from the cartilage of humans or large animals (e.g., chickens and horses) [8,9] are available, but this is not the case for Dunkin–Hartley guinea pigs, despite being the most appropriate model for studying idiopathic osteoarthritis [10–12]. While studies in smaller species such as mice often achieve high RNA quality, they frequently require pooling cartilage tissue from multiple animals to obtain sufficient material for extraction [13]. Moreover, advanced techniques such as single-cell RNA sequencing in mice also necessitate pooling cartilage from a minimum of five mice [14], which may not be feasible or align with certain experimental designs, especially in contexts where animal availability is restricted.

Owing to its limited cellular population and elevated proteoglycan content, the extraction of high-quality total RNA from cartilage poses a particular challenge. This makes gene expression analysis directly from the cartilage tissues technically demanding, yet critically important. High-quality RNA is essential not only for accurate gene expression profiling but also for exploring epigenetic mechanisms involved in OA, such as regulation by non-coding RNAs (e.g., microRNAs). Gene expression analysis provides a molecular snapshot of the disease state and allows for the identification of differentially expressed genes and signaling pathways involved in OA progression, inflammation, and cartilage degradation. These molecular signatures can help uncover key regulatory nodes that drive disease onset and advancement, thereby serving as targets for therapeutic intervention. While gene expression alone may not fully elucidate OA pathogenesis, it remains a foundational and indispensable tool in dissecting the molecular mechanisms of the disease and guiding the development of targeted, mechanism-based therapies.

Based on the importance of investigating gene expression within chondrocytes, the challenges associated with RNA isolation from small amounts of cartilage tissue, and the limitations of existing methodologies, we present an improved RNA isolation protocol in resource-limited setups from the articular cartilage and synovium of Dunkin–Hartley guinea pigs. The protocol has been designed to optimize sample collection and tissue processing procedures, aiming to minimize RNA degradation while increasing RNA quality. It has significant implications for advancing both basic OA research and translational applications. Our findings demonstrate that the RNA obtained from the articular cartilage and synovial tissues of Dunkin–Hartley guinea pigs using the optimized methodology is suitable for downstream applications such as RTqPCR. However, a few limitations remain. While the extracted RNA exhibited acceptable purity and concentration, the RNA integrity number (RIN) values were below the threshold generally required for high-throughput applications such as RNA sequencing or transcriptome profiling. Despite this, the RNA quality was sufficient for reliable RT–qPCR analysis, providing a practical and cost-effective alternative, particularly for resource-limited settings. Additionally, we observed variability in RNA yield from 12-month-old animals, likely due to increased OA severity and the corresponding reduction in the quantity of available tissue. This variability poses a challenge for reproducibility and consistency. Future refinements in tissue handling, preservation, and extraction techniques may be necessary to further enhance RNA quality and expand the applicability of this protocol to broader transcriptomic studies.

## Materials and reagents

**Reagents**

1. Liquid nitrogen

2. TRIzol^®^ reagent (Invitrogen, catalog number: 15596026 or equivalent)

3. RNaseZap or RNase AWAY for RNase decontamination

4. Chloroform (Himedia, catalog number: AS040 or equivalent)

5. Glycoblue^TM^ coprecipitant (Invitrogen, catalog number: AM9516)

6. Isopropanol (Himedia, catalog number: AS068 or equivalent)

7. Ethanol (Himedia, catalog number: MB106 or equivalent)

8. Nuclease-free water (Sigma, catalog number: 3098 or equivalent)

9. RNeasy^TM^ Mini kit (Qiagen, catalog number: 74104)

10. RNAqueous^TM^ kit (Invitrogen, catalog number: AM1912)

11. Quick-RNA^TM^ Miniprep Plus kit (Zymo Research, catalog number: R1057)

12. RNase-free DNase set (Qiagen, catalog number: 79254)

13. miRCURY LNA RT kit (Qiagen, catalog number: 339340)

14. miRCURY LNA SYBR Green PCR kit (Qiagen, catalog number: 339346)

15. Custom miRCURY LNA 5S rRNA PCR assay (Qiagen, catalog number: 339317, GeneGlobe ID: YCP0527201)

16. iScript cDNA Synthesis kit (Bio-Rad, catalog number: 1708891)

17. β-actin primers (Sigma-Aldrich, Merck, purification: desalt)

18. Trisodium citrate dihydrate (Himedia, catalog number: TC249)

19. Sodium chloride (Himedia, catalog number: MB023)

**Solutions**

1. 3 M sodium citrate (see Recipes)

2. 3 M sodium chloride (see Recipes)

3. 70% ethanol (see Recipes)

**Recipes**

**1. 3 M sodium citrate**

**Table:** 

Reagent	Final concentration	Quantity or volume
Trisodium citrate dihydrate (MW = 294.10 g/mol)	3 M	44.12 g (for 50 mL final volume)
Nuclease-free water	N/A	50 mL

Weigh 44.12g of trisodium citrate dihydrate and dissolve it in approximately 40mL of nuclease-free water. Stir the solution until completely dissolved, then adjust the final volume to 50mL with nuclease-free water. Store the solution at 4 °C.

**2. 3 M sodium chloride**

**Table:** 

Reagent	Final concentration	Quantity or volume
Sodium chloride (MW = 58.44 g/mol)	3 M	3.51 g (for 50 mL final volume)
Nuclease-free water	N/A	50 mL

Weigh 3.51g of sodium chloride and dissolve it in approximately 40mL of nuclease-free water. Stir the solution until completely dissolved, then adjust the final volume to 50mL with nuclease-free water. Store the solution at 4 °C.

**3. 70% ethanol**

**Table:** 

Reagent	Final concentration	Quantity or volume
Absolute ethanol (100%)	70% (v/v)	35 mL
Nuclease-free water	30% (v/v)	15 mL

**Laboratory supplies**

1. Stainless-steel vials (Biospec, catalog number: 2007)

2. 3.2 mm stainless-steel beads (Biospec, catalog number: 11079132ss)

3. 1.5 mL microcentrifuge tubes (Eppendorf tubes 3810X, catalog number: 0030125215)

4. 22G needles (Lifelong Safeway) and 1 mL syringe (Accu-Shot One, catalog number: 1A1R1P)

## Equipment

1. Mini-Beadbeater-16 (Biospec, catalog number: 607)

2. Centrifuge with a temperature range of up to 4 °C (ThermoFisher Scientific, catalog number: 75007213)

3. Micro-pipettes 0.5–10, 10–100, 20–200, and 100–1,000 μL (Eppendorf)

4. Benchtop centrifuge (ThermoFisher Scientific, catalog number: 75002414)

5. Laminar hood (Labtop Laminar Air Flow, product ID: 5116877-50624733960) or a clean and isolated working area

6. NanoDrop^®^-ND 1000 spectrophotometer (ThermoFisher Scientific, catalog number: ND-1000)

7. RT-qPCR (Bio-Rad, model: CFX96)

## Software and datasets

1. NanoDrop^®^ ND-1000 Software (v3.7.1, Thermo Fisher Scientific, 2015)

2. Bio-Rad CFX Manager Software (v3.1, Bio-Rad Laboratories, 2014)

3. Microsoft Excel

## Procedure

Sacrifice the guinea pigs in accordance with ethical standards and institutional guidelines. Dissect the knee joints by making a longitudinal incision through the skin and underlying muscle to expose the joint. Carefully remove surrounding soft tissues using sterile forceps and scissors, taking care not to damage the joint capsule. Gently displace the patella to access the joint cavity. Harvest the synovial membrane located in the suprapatellar region and along the inner surface of the joint capsule, just inferior to the patella, and around the femoral condyles. Following synovial tissue collection, cut the cruciate ligaments using a sterile surgical blade to fully expose the tibiofemoral compartment and articular cartilage. Wash the exposed joint area briefly with 70% ethanol prepared in nuclease-free water to reduce surface microbial contamination, thereby preserving sample integrity for downstream molecular analyses. After harvesting the cartilage and synovial tissues, immediately transfer them into 2 mL stainless steel vials containing three 3.2 mm stainless steel beads. Flash-freeze the tissues by immersing the vials in liquid nitrogen and storing them at -80 °C or in a liquid nitrogen tank until further processing.

**Caution:** In our experience, a maximum of only 10–20 mg of cartilage and synovial tissues can be obtained from one guinea pig knee joint, depending on the animal’s age, severity of OA, and degree of cartilage degeneration. Therefore, it is necessary to be extremely cautious when extracting and handling tissue samples.

**A. Tissue homogenization**

For **cartilage homogenization**, use a Mini-Beadbeater.

1. Immediately transfer the cartilage samples stored in stainless-steel vials (containing three 3.2 mm stainless steel beads) from -80 °C into liquid nitrogen before homogenization.

2. Perform 3–4 cycles of dry grinding for 15 s each.

3. After each cycle, cool the vials for 2 min by immersing them in liquid nitrogen to dissipate the heat generated.

**Caution:** Ensure that cartilage samples do not exceed liquid nitrogen temperatures during homogenization and keep them frozen until TRIzol^®^ (for Protocols 1 and 2, see below) or other appropriate lysis buffer (for Protocols 3, 4, and 5, see below) is added.

4. Add 500µL of ice-cold TRIzol^®^ reagent for Protocols 1 and 2, or the appropriate volume of lysis buffer for the other protocols: 300µL for Protocol 3, 140µL for Protocol 4, and 300µL for Protocol 5.

5. Perform two cycles of bead beating for 15 s each.

6. Cool the vials on ice for 2 min between each cycle to preserve RNA quality.

*Note: The number of bead-beating cycles and tissue grinding parameters may vary slightly based on the amount of cartilage tissue harvested from the knee joint, which mainly depends on the age of the animal and OA severity. The amount of synovial tissue remains relatively consistent regardless of the animal’s age or OA severity; therefore, no adjustments to the homogenization procedure for synovial tissue are necessary. Based on our experience, we have optimized the procedure as shown in Table 1.*

**Table 1.** Homogenization of cartilage tissues

Age of animals	Amount of harvested cartilage tissue	No. of homogenization cycles
3 months	Approximately 20 mg	4 cycles of dry-grinding, 2 cycles with buffer (15 s)
7 months	Approximately 12–15 mg	3 cycles of dry-grinding, 2 cycles with buffer (15 s)
12 months	Approximately 10 mg	3 cycles of dry-grinding, 1 cycle with buffer (15 s)

For **synovium homogenization**, use a Mini-Beadbeater.

1. Immediately transfer the synovium samples stored in stainless-steel vials (containing three 3.2 mm stainless steel beads) from -80 °C to ice before homogenization.

2. Add 500µL of ice-cold TRIzol^®^ reagent for Protocols 1 and 2 (see below), or the appropriate volume of lysis buffer for the other protocols: 300µL for Protocol 3, 140µL for Protocol 4, and 300µL for Protocol 5 (see below).

3. Perform 10 cycles of bead beating for 15 s each.

4. After each cycle, cool the vials on ice for 2 min to prevent RNA degradation.

**B. RNA isolation**

**Protocol 1: RNA isolation via the TRIzol^®^ method**

1. After homogenization, add another 500 μL of ice-cold TRIzol^®^ reagent to the stainless-steel vials, adjusting the total volume to 1 mL.

*Note: This protocol uses a total of 1 mL of TRIzol per ~20 mg of tissue. The volume of TRIzol can be adjusted based on the amount of tissue collected (e.g., 800µL for 10mg of tissue).*

2. Spin the vials briefly for 30 s and transfer the homogenate into RNase-free microcentrifuge tubes (MCTs).

3. Incubate the homogenate at room temperature for 5–10 min.

4. Centrifuge at 12,000× *g* for 5 min at 4 °C to pellet debris and ensure undigested particles are removed.

5. Transfer the clear supernatant into a new MCT in a laminar hood or RNase-free area.

***Modification 1***: *Pass the homogenate 10 times through a 22G needle to improve homogenization*.

6. Add 200 μL of chloroform, shake vigorously, and incubate at room temperature for 15 min.

*Note: TRIzol and chloroform are used at a 5:1 ratio. If using 800µL of TRIzol, adjust the chloroform volume proportionally (i.e., use 160µL of chloroform).*

7. Centrifuge at 12,000× *g* for 15 min at 4 °C to separate phases. Carefully transfer the upper aqueous layer into a new MCT.

8. Add 0.5 volumes of 100% isopropanol to the aqueous layer.

***Modification 2***: *Estimate sample volume using a pipette. Add concentrated 3 M sodium chloride and 3 M sodium citrate (prepared in nuclease-free water) to a final concentration of 1.2 M and 0.8 M, respectively, to improve RNA purity from proteoglycan-rich tissues.*

***Modification 3***: *Add 200 μL of chloroform again to the aqueous phase and repeat steps B6 and B7 to further remove phenol, guanidine, and salt contaminants.*

***Modification 4***: *Add ammonium acetate to 0.5 M, Glycoblue coprecipitant to 50 µg/mL, and 1 volume of 100% isopropanol. Incubate at -20 °C for 15 min to enhance RNA precipitation.*

***Modification 5***: *Alternatively, incubate the isopropanol mixture for 2–3 h or overnight at -20 °C to improve RNA yield*.

9. Precipitate RNA by centrifuging at 12,000× *g* for 15 min at 4 °C and discard the supernatant carefully.

10. Wash the RNA pellet twice with 1 mL of 75% ethanol, centrifuging at 8,000× g for 5 min at 4 °C after each wash to remove residual salts.

11. Air-dry the pellet at room temperature and resuspend in 20 μL of nuclease-free water.

**Caution:** Do not over-dry the RNA pellet, as this can make it difficult to resuspend. Air-dry at room temperature for approximately 5–10 min or until the RNA pellet appears slightly translucent and no visible liquid remains.

12. Quantify RNA concentration and purity using a NanoDrop system and store the RNA at -80 °C until further use.

**Protocol 2: RNA isolation via the TRIspin method** (TRIzol^®^/RNeasy^TM^)

1. After homogenizing the tissues as previously described (see section A) and following Protocol 1 up to step 7, add an equal volume of 100% ethanol to the aqueous phase and mix gently.

2. Load up to 680 μL of the sample onto the RNeasy^TM^ Mini column and discard the flowthrough. Repeat this step as needed based on the total sample volume.

***Modification***: *Perform on-column DNase treatment using the RNase-free DNase set according to the manufacturer’s instructions to remove genomic DNA contamination.*

3. Proceed with RNA isolation following the RNeasy™ Mini kit protocol provided by QIAGEN and elute the purified RNA in 20 μL of nuclease-free water.

*Note: The RNeasy^TM^ Mini kit user manual is available at https://www.qiagen.com/.*

**Protocol 3: RNA isolation via the RNeasy™ Mini kit**

1. Homogenize the samples as previously described (see section A). Add another 300 μL of RLT buffer to the stainless-steel vials, adjusting the total volume to 600 μL, optimized for 10–20 mg of cartilage and synovium tissues.

***Modification:*** *Add 6 μL of β-mercaptoethanol to every 600 μL of RLT buffer to enhance RNA protection by inactivating RNases, which is especially important for RNA isolation from tissues rich in RNases.*

2. Proceed with RNA isolation following the manufacturer’s instructions provided in the RNeasy™ Mini kit manual (Qiagen).

**Protocol 4: RNA isolation via the RNAqueous^TM^ kit**

1. Homogenize cartilage and synovium samples using lysis buffer from the RNAqueous^TM^ Kit as previously described (see section A). Add another 100 μL of lysis buffer to the stainless-steel vials, adjusting the total volume to 240 μL. This protocol uses lysis buffer at a ratio of 10–12 μL per milligram of tissue.

2. Transfer the homogenate to a new microcentrifuge tube. Perform RNA isolation following the manufacturer’s instructions.

*Note: The RNAqueous™ kit manual is available at https://www.thermofisher.com/*.

**Protocol 5: RNA isolation via the Quick-RNA^TM^ Miniprep Plus kit**

1. Homogenize synovium and cartilage tissues as previously described (see section A). Add another 300 μL of RNA Shield (from the Quick-RNA^TM^ Miniprep Plus kit) to the stainless-steel vials, adjusting the total volume to 600 μL, optimized for 10–20 mg of cartilage and synovium tissues. RNA Shield inactivates nucleases and is ideal for sample storage.

2. Transfer the homogenate into a new microcentrifuge tube inside a laminar hood or an RNase-decontaminated area.

***Modification:*** *Add 30 μL of Proteinase K mixed with 60 μL of Proteinase K digestion buffer to the homogenate. Incubate at room temperature for 3–4 h. Prepare Proteinase K by reconstituting the lyophilized enzyme in 1.04 mL of Proteinase K storage buffer.*

*Note: Although the addition of proteinase K is recommended in the manufacturer’s instructions for the Quick-RNA^TM^ Miniprep Plus kit, its inclusion in this protocol was found to be a critical step for improving RNA yield and purity from cartilage and synovium samples. Among all tested protocols, only this method incorporated proteinase K treatment, which significantly enhanced tissue digestion and RNA quality.*

3. Centrifuge at 12,000× *g* for 5 min at 4 °C to pellet undigested tissue.

4. Transfer the supernatant to a new 2 mL MCT and add an equal volume of RNA lysis buffer. Mix gently by pipetting.

5. Transfer the sample into a yellow Spin-Away™ filter in a collection tube. Centrifuge at 11,000× *g* for 30 s.

*Note: This step removes most genomic DNA.*

6. **Save the flowthrough** and repeat step 5 if needed based on sample volume.

7. Add an equal volume of 100% ethanol to the flowthrough and mix gently.

8. Load the mixture onto a Zymo-Spin^TM^ IIICG column (green) and centrifuge at 11,000× *g* for 30 s. Repeat until all the sample passes through.

9. Perform DNase I digestion:

a. Wash the column with 400 μL of RNA wash buffer, centrifuge at 11,000× *g* for 30 s, and discard the flowthrough.

b. Add 5 μL of DNase I mixed with 75 μL of DNA digestion buffer directly to the column. Incubate for 15 min at room temperature.

10. Add 400 μL of RNA prep buffer, centrifuge at 11,000 × *g* for 30 s, and discard the eluate.

11. Wash the column with 680 μL of RNA wash buffer, centrifuge at 11,000× *g* for 30 s, and discard the flowthrough.

12. Perform another wash with 400 μL of RNA wash buffer and centrifuge at 11,000× *g* for 1 min.

13. Transfer the column to a new nuclease-free 1.5 mL tube and elute RNA:

a. Add 10 μL of nuclease-free water, incubate for 10 min, and centrifuge at 11,000× *g* for 1 min.

b. Perform a second elution with 5 μL of nuclease-free water, incubate for 5 min, and centrifuge at 11,000× *g* for 30 s.

c. Perform a final dry spin for 45 s at 8,000 × g to remove residual wash buffer.

**Critical:** Using a smaller volume of nuclease-free water and eluting twice helps increase the RNA yield.

**C. QC of RNA samples**

1. Quantify RNA concentration and assess purity using a NanoDrop^®^ ND-1000 spectrophotometer. Ensure the instrument is blanked with nuclease-free water before use. The small sample requirement makes it ideal for measuring RNA isolated from limited amounts of tissue, where only a limited quantity of input RNA is available.

2. The A_260:280 _ratio is a measure of the purity of RNA samples. A high A_260:280 _ratio (1.8–2.0) indicates that the RNA sample is relatively free from protein contamination, making it suitable for downstream applications.

3. The A_260:230 _ratio is another indicator of RNA sample purity, but it specifically reflects the presence of contaminants such as phenols, chaotropic salts, and other organic compounds that may copurify with RNA during extraction. A high A_260:230 _ratio (>1.7) indicates minimal contamination by organic compounds.

**D. RTqPCR to assess RNA quality**

To evaluate the impact of RNA quality on gene expression, RT–qPCR can be used to detect the expression levels of endogenous control genes, specifically 5S rRNA and ß-actin.

1. Synthesize cDNA for 5S rRNA from the extracted RNA (approximately 100 ng) using the miRCURY LNA RT kit, following the manufacturer’s instructions.

2. Perform RT–qPCR for 5S rRNA detection using the miRCURY LNA SYBR Green PCR kit and a custom miRCURY LNA PCR assay designed specifically for guinea pig 5S rRNA.

a. Run reactions in duplicate for each sample.

b. Thermal cycling conditions: Initial denaturation at 95 °C for 2 min, 40 cycles of denaturation at 94 °C for 10 s and annealing/extension at 56 °C for 60 s.

3. Synthesize cDNA from the extracted RNA (approximately 300–400 ng) for ß-actin using the iScript cDNA Synthesis kit according to the manufacturer’s protocol.

4. Perform RT–qPCR for ß-actin expression using the following primer sequences:

- Forward: TAACTTGCGCGCAGAAAACG
- Reverse: GTCCTCGGCCACATTGTAGA

5. Include NO-RT (no reverse transcription) controls for each RNA sample to assess genomic DNA contamination.

a. Compare the Ct values of cDNA and NO-RT reactions.

b. A ΔCt ≥ 5 indicates effective removal of gDNA and confirms RT specificity.

6. Validate PCR specificity using melt curve analysis to confirm the presence of a single amplification peak for each reaction.

7. Use real-time PCR software (e.g., Bio-Rad CFX Manager) to analyze amplification and melt curves.

*Note: To confirm the absence of genomic DNA contamination in the reverse-transcribed RNA samples, isolated via the Quick-RNA™ Miniprep Plus kit, the results of RTqPCR with ß-actin were compared with those of the corresponding NO-RT (no reverse transcription) controls. The amplification curves* ***(Dataset S2)*** *for the cDNA samples showed early amplification, and the NO-RT controls displayed amplification at much higher Ct values. The observed ΔCt of 5.8–10.2 cycles between the cDNA samples and NO-RT controls confirms a ~100-fold difference in template quantity, confirming the effective removal of genomic DNA contamination.*

## Data analysis

**A. Spectrophotometric comparison of RNA protocols**

**Table 2.** Comparative analysis of methods performed for RNA isolation from guinea pig cartilage and synovium tissues

Method	Tissue	Modification		Nanodrop parameters		
			RNA conc. (ng/µL)	Yield (μg)	A_260:280_	A_260:230_
a) TRIzol^®^	Cartilage	Homogenate extruded through a 22G needle 10 times, and addition of 1.2 M sodium chloride + 0.8 M sodium citrate to the aqueous layer	1447	28.94	1.07	0.47
		Homogenate extruded through a 22G needle 10 times and overnight isopropanol incubation (-20 °C)	235.3	4.7	1.39	0.39
	Synovium	Homogenate extruded through a 22G needle 10 times and overnight isopropanol incubation (-20 °C)	115.7	2.31	1.43	0.26
		Second chloroform extraction	11.1	0.22	1.57	0.22
		Glycogen coprecipitation using Glycoblue^TM^	105	1.05	1.45	0.53
b) TRIspin	Cartilage	On-column DNase treatment	57.55	1.15	1.5	0.24
	Synovium	Second chloroform extraction	78.5	1.57	1.45	1.5
c) RNeasy^TM^	Cartilage	On-column DNase treatment	22.3	0.44	1.79	-
	Synovium		23.4	0.46	1.63	0.74
d) RNAqueous^TM^	Cartilage	-	6.1	0.24	1.59	-
	Synovium		11.7	0.46	1.42	0.06
e) Quick-RNA^TM^ Miniprep Plus Kit	Cartilage	Proteinase K treatment	106.3	2.12	2.0	1.62
	Synovium		265.1	5.3	1.98	2.01

The prerequisite for good-quality RNA isolation from animal cartilage and synovial tissues is the use of an appropriate homogenization method to ensure the extraction of RNA from highly crosslinked protein-rich tissues. We used bead beating for the homogenization of both cartilage and synovial tissues and modified the number of bead-beating cycles according to the initial amount of tissue to avoid harsh homogenization, which can degrade the RNA. Liquid nitrogen or ice was used between cycles to dissipate the heat generated. RNA was isolated via the traditional TRIzol^®^ method, and the incorporation of different modifications resulted in low RNA quality, as indicated by the low A_260:280 value _(1.07–1.57). In contrast to the results of Bleu et al., who demonstrated the need for high salt concentrations to extract high-purity RNA from cartilage tissues with high proteoglycan contents [4], the addition of 1.2 M sodium chloride and 0.8 M sodium citrate to the aqueous layer (*Modification 2*) resulted in low A_260:280 _and A_260:230 _ratios **(Table 2)**. A low A_260:230 _ratio indicates high salt contamination. The second chloroform isolation (*Modification 3*) of synovium samples, which is usually performed to overcome the problem of RNA contamination due to phenol, guanidine, and salts, is common in the conventional phenolchloroform-based RNA isolation method, and drastically reduces the RNA concentration but results in a better A_260:280 _ratio. Moreover, no notable improvement in the RNA concentration or purity was observed with the use of Glycoblue *(Modification 4)*, which facilitates RNA recovery [15,16]. We also used the TRIspin method (TRIzol^®^/RNeasy^TM^), a combination of the traditional TRIzol^®^ and RNeasy^TM^ kit methods, to isolate RNA from cartilage and synovial tissues. Although the on-column DNase treatment (*Modification 5*) resulted in an improved A_260:280 _ratio, the A_260:230 _ratio remained low. In contrast to the low A_260:230 _ratio observed in the cartilage samples isolated via the TRIspin method, the RNA isolated from the synovium samples via this method, along with the addition of a second chloroform extraction, exhibited a prominent increase in the A_260:230 _ratio.

There are numerous disadvantages of using the traditional phenol/chloroform-based method to extract RNA from extremely small amounts of tissue, such as low visibility of the RNA pellet and low purity due to phenol or gDNA contamination. The spin column–based methods for RNA isolation provide great scope for yielding high-quality RNA within a short processing time. The RNA isolated from cartilage and synovial tissues via the RNeasy^TM^ Mini kit resulted in A_260:280 _ratios of 1.79 and 1.63, respectively, but the RNA concentration obtained was too low. The RNAqueous^TM^ kit–based method for RNA isolation results in extremely low RNA concentrations and A_260:230 _ratios, making it unsuitable for further downstream applications such as RT–qPCR. Compared with all the methods used for RNA isolation from guinea pig cartilage and synovial tissues, the Quick-RNA^TM^ Miniprep Plus kit–based method **(Protocol 5)**, which involves proteinase K treatment, resulted in the highest RNA concentration **(Dataset S1)** along with high A_260:280 _and A_260:230 _ratios. Proteinase K treatment effectively facilitated the degradation of proteins and nucleases. These results indicate the suitability of the Quick-RNA^TM^ Miniprep Plus kit for extracting high-quality RNA from small amounts of guinea pig cartilage and synovium samples.

**B. Gene expression for RNA QC**

**Figure 1.**
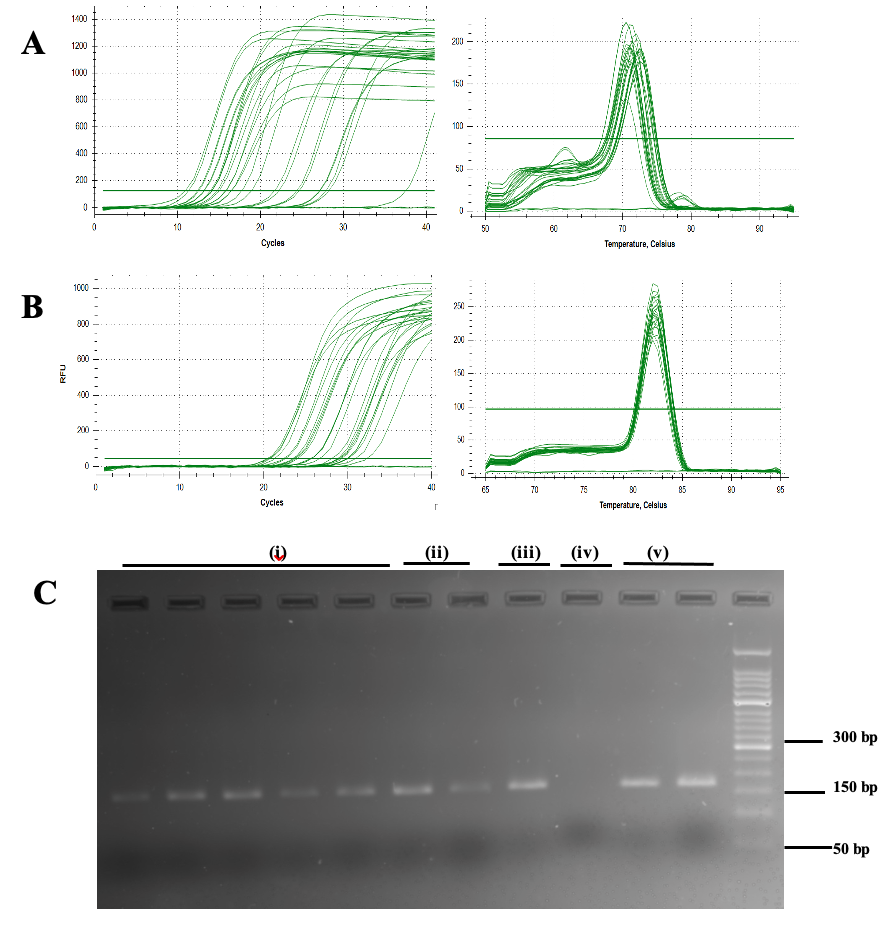
Real-time quantitative PCR amplification and melt curve plot. (A) 5S rRNA and (B) β-actin genes in cartilage and synovium tissues. **(C)** Visualization of PCR products of ß-actin (amplicon length: 170 bp); i) TRIzol^®^; ii) TRIspin; iii) RNeasy^TM^; iv) RNAqueous^TM^; and v) Quick-RNA^TM^ Miniprep Plus kit in 2% agarose gel stained with ethidium bromide.

RT–qPCR, which was conducted to evaluate the influence of RNA quality on gene expression **(Figure 1)**, revealed that the method utilized for RNA extraction had a discernible influence on the outcomes of the gene expression assays. The results demonstrated that the RNA isolated via methods other than the Quick-RNA^TM^ Miniprep Plus kit consistently required a greater number of amplification cycles (higher C_T_ values) for the detection of 5S rRNA and ß-actin **(Table 3)**. The Protocol 5 with Quick-RNA^TM^ Miniprep Plus kit presents an optimized RNA isolation method for small amounts of guinea pig cartilage and synovium, designed for RT–qPCR in resource-limited settings. While the RIN values were below the threshold **(Dataset S1)** for high-throughput applications such as RNA sequencing or transcriptome profiling, the extracted RNA was sufficient for RT–qPCR, providing a practical and cost-effective alternative. Future refinements are needed to enhance RNA quality for broader applications, but this method represents a valuable tool for gene expression studies in challenging tissue types.

**Table 3.** RNA extraction method’s influence on the outcome of gene expression assays via RT–qPCR

Method	Tissue	CT values (real-time PCR)	
a) TRIzol®		5S rRNA	ß-actin
	Cartilage	14.22	27.45
		16.46	26.71
	Synovium	14.86	26.77
		24.48	31.07
		21.91	28.32
b) TRIspin	Cartilage	11.09	24.8
	Synovium	28.04	28.63
c) RNeasy^TM^	Cartilage	NA	NA
	Synovium	15.78	24.9
d) RNAqueous^TM^	Cartilage	*	*
	Synovium		
e) Quick-RNA^TM^ Miniprep Plus Kit	Cartilage	12.44	24.30
	Synovium	14.03	22.99

## Validation of protocol

This protocol is a part of our ongoing research work on deciphering the role of miRNAs in the pathogenesis of idiopathic OA in a guinea pig model. To ensure the reliability of our method, we performed RNA isolation from synovium and cartilage samples multiple times throughout our experiments involving gene expression studies. These repeated isolations consistently yielded RNA suitable for RT–qPCR, demonstrating the reproducibility of our protocol. The protocol discusses the critical role of homogenization in successful RNA isolation from cartilage and synovium tissues. A detailed description of the homogenization protocol, including the number of bead-beating cycles, tissue grinding time, and cooling intervals, is provided in the methods section. These parameters were optimized through repeated experiments to ensure efficient tissue disruption while avoiding RNA disruption. The protocol will be cited accordingly in future publications. As detailed in **Dataset S1**, the RNA yield and purity from cartilage samples isolated at different time points were consistent. RNA concentrations ranged from 65 to 240 ng/µL, depending on the age and severity of OA in the animals, which influences the amount of cartilage obtainable from the knee joints. The purity of the RNA, assessed by A_260:280_ ratios, consistently ranged from 1.7 to 2.0. While our repeated isolations have demonstrated reproducibility, further assessments involving independent operators or laboratories could strengthen these findings.

## General notes and troubleshooting

**General notes**

1. All laboratory benches, pipettes, and dissection tools must be cleaned using an RNase decontamination solution such as RNaseZap or RNase AWAY.

2. Only RNase-free or autoclaved and DEPC-treated microcentrifuge tubes and pipette tips should be used throughout the procedure to prevent RNA degradation.

3. All equipment and solutions that come in contact with the cartilage and synovial tissues must be sterile and free from RNase contamination.

4. Only stainless-steel vials must be used for bead beating, as the milling forces can crack common plastic micro-vials and support faster sample cooling steps.

5. The stainless-steel vials used for tissue storage must be autoclaved and then rinsed with chloroform before usage. Chloroform must be completely evaporated before tissue contact.

6. The tissues must be flash-frozen within 2–3 min of removal from the guinea pig knee joint in the operating room to minimize RNA degradation.

7. Stainless steel vials must be placed on ice throughout the process of cartilage tissue excision and sample collection.

8. The tissues must be snap-frozen for at least 2 min before storing at -80 °C.

9. During work with animals, gloves, appropriate personal protective equipment (PPE), and a face mask must be worn.

10. The gloves must be changed frequently while harvesting the cartilage and synovial tissues to avoid cross-contamination.

11. RNase inhibitor solutions such as RNAlater^®^ must not be used to store or transport cartilage and synovial tissues, as they can cause dehydration [5], leading to hardening of the tissues and, subsequently, making the homogenization process difficult.

12. A maximum of only 10–20 mg of cartilage and synovial tissues can be obtained from one guinea pig knee joint, depending on the animal’s age, severity of OA, and degree of cartilage degeneration. Therefore, it is necessary to be extremely cautious when extracting and handling tissue samples.

## Supplementary information

The following supporting information can be downloaded here:

1. Dataset S1. QC of RNA isolated from cartilage using the Quick-RNA™ Miniprep Plus Kit

2. Dataset S2. RNA Sample Purity by NO-RT Controls
